# *NLRP3* Influences Cognitive Function in Schizophrenia in Han Chinese

**DOI:** 10.3389/fgene.2021.781625

**Published:** 2021-12-10

**Authors:** Ruimei Liu, Wei Tang, Weiping Wang, Feikang Xu, Weixing Fan, Yi Zhang, Chen Zhang

**Affiliations:** ^1^ Schizophrenia Program, Shanghai Mental Health Center, Shanghai Jiao Tong University School of Medicine, Shanghai, China; ^2^ Department of Psychiatry, The Affiliated Kangning Hospital of Wenzhou Medical University, Wenzhou, China; ^3^ Department of Psychiatry, Jinhua Second Hospital, Jinhua, China; ^4^ Shanghai Key Laboratory of Psychotic Disorders, Shanghai Mental Health Center, Shanghai Jiao Tong University School of Medicine, Shanghai, China

**Keywords:** *NLRP3*, cognitive impairment, schizophrenia, RBANS, polymorphism

## Abstract

It has been proposed that immune abnormalities may be implicated with pathophysiology of schizophrenia. The nod-like receptor pyrin domain-contraining protein 3 (NLRP3) can trigger immune-inflammatory cascade reactions. In this study, we intended to identify the role of gene encoding NLRP3 (*NLRP3*) in susceptibility to schizophrenia and its clinical features. For the *NLRP3* mRNA expression analysis, 53 drug-naïve patients with first-episode schizophrenia and 56 healthy controls were enrolled. For the genetic study, a total of 823 schizophrenia patients and 859 controls were recruited. Among them, 239 drug-naïve patients with first-episode schizophrenia were enrolled for clinical evaluation. There is no significant difference in *NLRP3* mRNA levels between patients with schizophrenia and healthy controls (*p* = 0.07). We did not observe any significant differences in allele and genotype frequencies of rs10754558 polymorphism between the schizophrenia and control groups. We noticed significant differences in the scores of RBANS attention and total scores between the patients with different genotypes of rs10754558 polymorphism (*p* = 0.001 and *p* < 0.01, respectively). Further eQTL analysis presented a significant association between the rs10754558 polymorphism and *NLRP3* in frontal cortex (*p* = 0.0028, *p* = 0.028 after Bonferroni correction). Although our findings did not support *NLRP3* confer susceptibility to schizophrenia, *NLRP3* may be a risk factor for cognitive impairment, especially attention deficit in this disorder.

## Introduction

Schizophrenia is a deliberating and severe neuropsychiatric disease with a cluster of psychotic symptoms and cognitive impairment. Early studies have documented that maternal infection during pregnancy is implicated with increased risk of schizophrenia in the offspring (Brown and Derkits, 2010). It is known that certain clinical and genetic features are sheared between some autoimmune diseases and schizophrenia (Benros et al., 2011). Therefore, it has been proposed that immunologic abnormalities is likely to be involved in the development of schizophrenia (Tomasik et al., 2016).

Evidence from genetic studies have indicated that the concordance rates of schizophrenia for monozygotic twins are around 40–50%, and its heritability is estimated approximately 80% (Sullivan et al., 2003). As such, genetic studies of schizophrenia may provide some clues to uncover the biological mechanism of this disorder (Dhindsa and Goldstein, 2016). Several genome-wide association studies (GWASs) have consistently reported that schizophrenia susceptibility genes are located in the region of extended human major histocompatibility complex (MHC) (Shi et al., 2009; Stefansson et al., 2009). A recent meta-analysis on postmortem brain studies showed a prominent increase in the expression of pro-inflammatory genes in schizophrenia patients (van Kesteren et al., 2017). Our previous studies have identified the important roles of complement factors and pro-inflammatory cytokines in the pathophysiology of schizophrenia (Ni et al., 2015; Wang et al., 2015; Zhang et al., 2017a; Zhang et al., 2017b; Zhang et al., 2018). These results strengthened the hypothesis of immune-inflammatory processes in the pathogenesis of schizophrenia.

Based on this hypothesis, maternal immune activation (MIA) animal model is used to mimick schizophrenia-like pathological, neurochemical changes and behaviors in rodents (Zuckerman and Weiner, 2005; Wolff et al., 2011). Recent studies demonstrated that nod-like receptor pyrin domain-contraining protein 3 (NLRP3) inflammasome is involved in the schizophrenia-like behaviors of MIA model (Ventura et al., 2020; Talukdar et al., 2021). The NLRP3 inflammasome plays a key role in the innate immune system that mediates pro-inflammatory cytokines (Ventura et al., 2020). The involvement of NLRP3 in major psychiatric disorders has been reported in patients with major depressive disorder (Alcocer-Gomez et al., 2014; Tian et al., 2021) and bipolar disorder (Kim et al., 2016; Scaini et al., 2019). However, the relationship of NLRP3 with schizophrenia is not well known.

The human gene encoding NLRP3 (*NLRP3*) is located on chromosome 1q44, where linkage findings were reported in schizophrenia (Saviouk et al., 2005). To the best of our knowledge, there is no genetic study to identify the role of *NLRP3* in schizophrenia. In this study, we hypothesized that *NLRP3* may confer susceptibility to schizophrenia. First, we examined the difference of *NLRP3* mRNA expression between schizophrenia patients and healthy controls. Rs10754558 is a functional single-neucleotide polymorphism (SNP) and mapped in the 3′-untranslated region of *NLRP3.* Evidence showed that this SNP influences the stability of *NLRP3* mRNA (Hitomi et al., 2009). Thus, we secondly sought to characterize the association of *NLRP3* rs10754558 polymorphism with schizophrenia in Han Chinese. Finally, we planned to conduct an eQTL (expression quantitative trait loci) analysis to detect the role of the rs10754558 polymorphism in *NLRP3* mRNA expression in brain.

## Methods

### Subjects

We recruited schizophrenia patients from three mental institutions in Eastern China, including Jinhua Wenzhou Kangning Hospital, Jinhua Second Hospital and Shanghai Mental Health Center. The criteria of patients with schizophrenia have been documented in our previous studies (Zhang et al., 2016a; Wang et al., 2016; Lu et al., 2017; Wang et al., 2020). In brief, criteria are as followings: 1) met the Diagnostic and Statistical Manual of Mental Disorders, Fourth Edition (DSM-IV) criteria for schizophrenia; 2) had a stable condition for more than 6 months prior to participate in this study; 3) had no physical illness or other psychiatric disorders. The healthy subjects were hospital staff and students in local medical schools, who have no physical or psychiatric diseases. All subjects were Han Chinese.

For the *NLRP3* mRNA expression analysis, 53 drug-naïve patients with first-episode schizophrenia and 56 healthy subjects were included. For the genetic study, a total of 823 schizophrenia patients and 859 controls were recruited. Among them, 239 drug-naïve patients with first-episode schizophrenia were enrolled for clinical evaluation. Detailed information on the participants was previously described (Zhang et al., 2014).

### Clinical Evaluation

The Positive and Negative Syndrome Scale (PANSS) was used to evaluate the severity of psychotic symptoms in schizophrenia patients. There is a correlation coefficient greater than 0.8 for the PANSS total scores in repeated assessments (Zhu et al., 2015). The Repeatable Battery for the Assessment of Neuropsychological Status (RBANS) was used to evaluate cognitive performance in this study (Randolph et al., 1998).

RNA extraction and Quantitative real-time polymerase chain reaction (PCR)

The processes of fasting peripheral blood extraction, total RNA extraction, reverse transcription reaction and RT-PCR were documented in our previous study (Zhang et al., 2016b).

### Analysis of Brain NLRP3 mRNA Expression

We detected the difference of *NLRP3* expression in brain between schizophrenia patients and healthy subjects through SZDB database, a newly developed on-line platform for schizophrenia research (http://www.szdb.org/) (Wu et al., 2017).

### Genotyping

SNP rs10754558 was genotyped using Improved Multiplex ligase Detection Rection (iMLDR) method, with technical support from Center for Human Genetics Resarch, Shanghai Genesky Biotech Co., Ltd.

### PGC Data Analysis

We extracted the schizophrenia genetic data from the Psychiatric Genomics Consortium (PGC, http://www.broadinstitute.org/mpg/ricopili/) database and reanalyzed the data set as an independent sample to validate our genetic results.

### Brain eQTL Analysis

It is known that schizophrenia is derived from abnormalities in brain. As such, we used the brain eQTL database (http://peana-od.inf.um.es:8080/UKBECv12/) for eQTL analysis of rs10754558 polymorphism in brain.

### Statistical Analysis

SHEsisplus (Shen et al., 2016) software was used to calculate the Hardy-Weinberg equilibrium (HWE) and compare differences of genotype and allele distribution between schizophrenia and control groups. ANCOVA was conducted to compare the difference of *NLRP3* mRNA expression between schizophrenia patients and healthy controls, with covariates such as gender, smoking and age controlled in the model. Statistical significance of differences in clinical features between genotype groups was compared using ANCOVA with covariates (e.g., age, gender, course of disease, treatment duration and doses) to minimize their potential effects. Power analysis was carried out by using the Quanto software (http://hydra.usc.edu/GxE). SPSS 17.0 (SPSS, Inc. Chicago, IL, United States) was used to conduct all the statistical calculation. *p* values were two-tailed, and *p* < 0.05 means statistically significant. Bonferroni correction was performed during multiple comparison testing.

## Results

**FIGURE 1:**
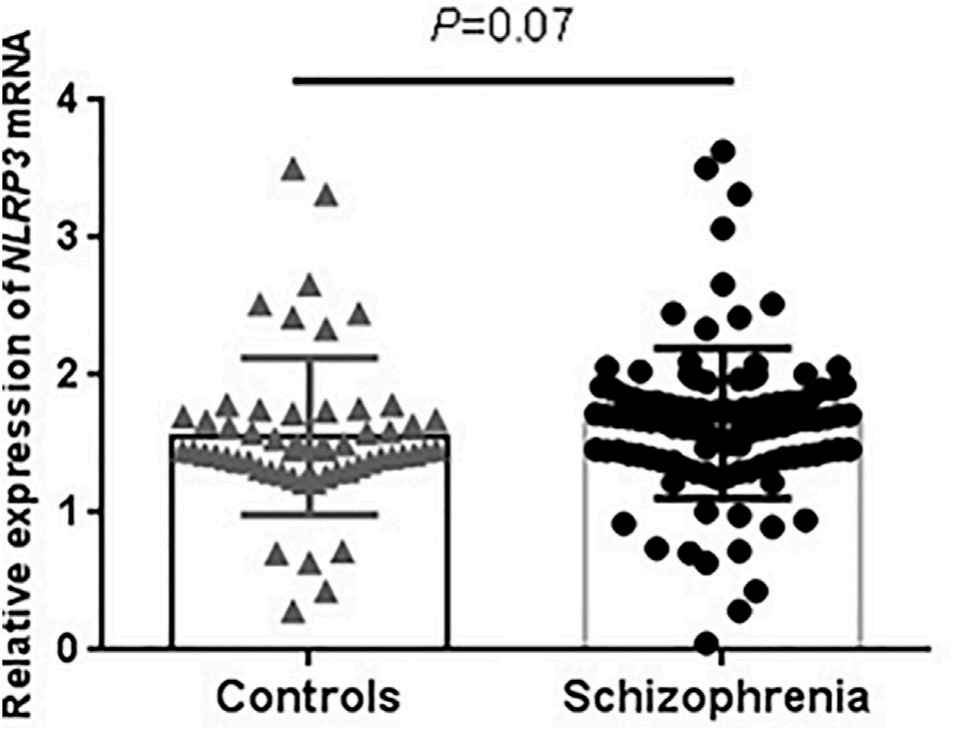
Peripheral expression levels of *NLRP3* mRNA in drug-naïve patients with first-episode schizophrenia and healthy controls. *NLRP3* mRNA level was normalized to that of GAPDH. Schizophrenia group (*n* = 53); Control group (*n* = 56).

For the expression analysis, demographic characteristics of our sample were presented in Supplementary Table S1. Schizophrenia and control groups are well-matched with respect to age, sex and smoking. The RT-PCR results showed no significant difference in *NLRP3* mRNA levels between schizophrenia and control groups (*p* = 0.07, Figure 1). We extracted brain *NLRP3* mRNA expression data from SZDB database. We did not observe significant differences of *NLRP3* mRNA expression in prefrontal cortex, hippocampus and striatum between schizophrenia patients and healthy controls (Supplementary Figure S1).

For the genetic analysis, we enrolled 823 schizophrenia patients and 859 healthy controls in this study. Demographic characteristics are well matched in both groups. No deviation from HWE was observed in genotype distribution for rs10754558 polymorphism in control group. The results of rs10754558 polymorphism were presented in Table 1. No significant differences in allele and genotype frequencies were found between the schizophrenia and control groups. With false positive rate controlled as 0.05, the statistical power to detect the odds ratio (OR) value as 1.5 for risk allele was expected to be more than 85% in our samples under the log additive model. We further examined the genetic association between the rs10754558 polymorphism and schizophrenia in PGC database, and we also did not observe significant association of rs10754558 polymorphism with schizophrenia (Supplementary Figure S2).

**TABLE 1:** Distribution of rs10754558 genotypes and alleles in schizophrenia patients and healthy controls.

SNP rs10754558	N	Genotype, N (%)			*P* ^a^	Allele, N (%)		*P* ^a^	OR (95%CI)	*P* *^b^*
		G/G	G/C	C/C		G	C			
Case	823	292 (35.4)	388 (47.1)	143 (17.3)	0.17	972 (59.0)	674 (40.9)	0.06	0.87 (0.76–1.01)	
Control	859	273 (31.7)	412 (47.9)	174 (20.2)		958 (55.7)	760 (44.2)			0.67

a Raw *p*-values.

b Hardy-Weinberg *p*-values.

**FIGURE 2:**
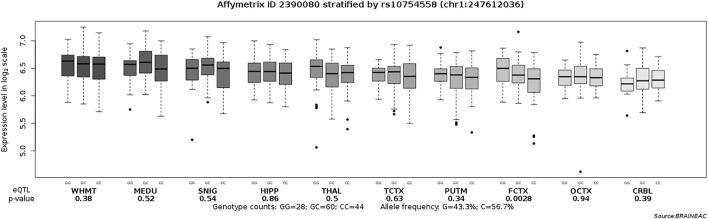
Association of rs10754558 polymorphism with the *NLRP3* mRNA expression level in ten brain regions (Affymetrix ID 2390080). SNIG, substantia nigra; PUTM, putamen (at the level of the anterior commissure); MEDU, the inferior olivary nucleus (sub-dissected from the medulla); THAL, thalamus (at the level of the lateral geniculate nucleus); OCTX, occipital cortex; HIPP, hippocampus; FCTX, frontal cortex; TCTX, temporal cortex; WHMT, intralobular white matter; CRBL, cerebellar cortex. Data were extracted from the BRAINEAC database (http://peana-od.inf.um.es:8080/UKBECv12/).

There were 239 drug-naïve patients with first-episode schizophrenia participated in the clinical evaluation. We examined the association of rs10754558 polymorphism with psychotic symptoms and cognitive performance in schizophrenia patients by comparing scores of the PANSS and RBANS with genotypes of SNP rs10754558. We observed significant differences in the scores of RBANS attention and total scores between the patients with different genotypes of rs10754558 polymorphism (*p* = 0.001 and *p* < 0.01, respectively), as shown in Table 2. Post-hoc comparisons showed that rs10754558 G/G or G/C carriers had lower RBANS attention and total scores than those with C/C carriers (*P*s<0.01). Subsequently, we conducted an eQTL analysis to test the influence of rs10754558 polymorphism on *NLRP3* mRNA expression in brain. Figure 2 showed that carriers with G allele have higher levels of *NLRP3* expression than those without G allele in frontal cortex.

**TABLE 2:** PANSS and RBANS performance comparisons of rs10754558 genotypic groups in patients with schizophrenia.

PANSS	G/G (*n* = 46)	G/C (*n* = 104)	C/C (*n* = 89)	*F* ^a^	*P* ^b^	*P* ^c^
Positive	25.87 ± 3.18	26.16 ± 2.98	26.40 ± 3.12	0.44	0.65	
Negative	17.43 ± 2.98	18.38 ± 2.71	18.09 ± 3.04	1.66	0.19	
General	41.85 ± 6.29	40.41 ± 6.09	41.21 ± 6.57	0.84	0.43	
Total score	85.15 ± 6.72	84.95 ± 7.58	85.71 ± 7.19	0.26	0.77	
RBANS						
Immediate memory	63.65 ± 6.92	65.53 ± 4.15	66.25 ± 2.62	4.73	0.01	0.1
Visuospatial skill	57.09 ± 9.40	58.84 ± 5.65	60.28 ± 5.36	3.62	0.03	0.3
Language	56.89 ± 5.12	55.89 ± 5.57	57.35 ± 4.86	1.95	0.14	
Attention	66.26 ± 13.60	70.61 ± 13.56	76.55 ± 20.34	7.21	0.001	0.01
Delayed memory	65.85 ± 9.78	67.10 ± 9.24	70.09 ± 11.35	3.19	0.04	0.4
Total score	309.74 ± 26.23	317.96 ± 22.15	330.52 ± 27.17	12.43	<0.01	<0.01

Data presented as *x* EQ ± s.

a *F* values adjusted for age, sex, years of education and duration of illness.

b Raw *p*-values.

c Adjusted *p*-values after Bonferroni correction.

## Discussion

There is a growing body of evidence implying that schizophrenia is attributed to a dysregulation of innate and adaptive immune system (Michel et al., 2012). Our previous studies have provided supportive evidence for the involvement of innate immune response in the pathogenesis of schizophrenia (Ni et al., 2015; Wang et al., 2015; Zhang et al., 2017b). NLRP3 is an intracellular protein involved in the initiation of the innate immune response (Bonar et al., 2012). It is known that *NLRP3* mutation confers susceptibility to immune disorders (Pontillo et al., 2010). Accordingly, we employed this study to identify the role of *NLRP3* in schizophrenia.

In this study, we found that peripheral *NLRP3* mRNA level has no difference between patients with schizophrenia and healthy controls. We also did not observe the difference of *NLRP3* mRNA level in brain between schizophrenia and controls. It has been well-established that MIA is associated with an increased risk of schizophrenia in offspring (Scola and Duong, 2017). Recent preclinical studies have found that activation of NLRP3 when LPS counts may be one of the potential mechanisms driving schizophrenia-like behavior in offspring in MIA model (Ventura et al., 2020; Talukdar et al., 2021). However, a postmortem study compared the expression of NLRP3-related protein in brain tissue from patients with schizophrenia and bipolar disorder in which increased level of NLRP3 was observed in patients with bipolar disorder, but not schizophrenia (Kim et al., 2016). Another comparative study also showed that peripheral level of NLRP3 is increased in patients with bipolar disorder, but not schizophrenia (Garcia-Alvarez et al., 2018). As such, our findings implied that *NLRP3* mRNA level is not a risk factor for schizophrenia.

Another aim of this study is to detect the association of *NLRP3* functional SNP rs10754558 with schizophrenia. SNP rs10754558 is located in the 3′-untranlated region (3′-UTR) of *NLRP3* and affects expression of *NLRP3* mRNA through altering its stability (Hitomi et al., 2009). This SNP has been reported to confer susceptibility to disorders of the central nervous system, such as Alzheimer’s disease (Tan et al., 2013), multiple sclerosis (Imani et al., 2018) and ischemic stroke (Lv et al., 2020). However, our results did not support the significance for rs10754558 polymorphism in the susceptibility to schizophrenia in either Han Chinese or European ancestry from PGC GWAS. This suggests that *NLRP3* SNP rs10754558 is probably not a risk SNP for schizophrenia.

Schizophrenia is known to have variable clinical presentation. Therefore, we further sought to determine the role of rs10754558 in the expression of clinical features in a sample of drug-naïve patients with first-episode schizophrenia. We observed a significant association of rs10754558 polymorphism with RBANS attention and total scores in such patients, but not psychotic symptoms. This suggested that rs10754558 polymorphism may result in cognitive impairment, especially attention deficit in schizophrenia. We further carried out an eQTL analysis to detect the effect of rs10754558 polymorphism on *NLRP3* mRNA expression in brain. Data showed that rs10754558 polymorphism is significantly associated with the *NLRP3* mRNA expression in frontal cortex, and the risk allele G is associated with increased level of *NLRP3* mRNA. There is accumulating evidence from magnetic resonance imaging (MRI) studies showing that frontal regions are critical for cognitive impairment in schizophrenia (Glascher et al., 2019). Our recent findings indicated that dysfunctional connectivity of frontal cortices is associated with cognitive impairment in patients with schizophrenia (Yu et al., 2021). North et al. (2021) found that elevated level of peripheral inflammation is a risk factor for cognitive deficits and brain structure in schizophrenia, especially reduced attention and cortical thickness. Meta-analysis also showed that attention deficit has a strongest relationship with peripheral inflammation in all cognitive domains tested in patients with schizophrenia (Bora, 2019). Our previous studies have demonstrated that elevated levels of pro-inflammatory cytokines are associated with cognitive impairment in schizophrenia (Zhang et al., 2017a; Zhang et al., 2018; Xu et al., 2019). NLRP3 is a critical regulator of pro-inflammatory cytokines production by activated caspase-1 to initiating an inflammatory form of cell death (Feng et al., 2021). Recent literature documented that enriched NLRP3 may lead to cognitive deficits, and inhibition of NLRP3 could ameliorate such impairment (Fang et al., 2021). As such, our results implied that increased level of *NLRP3* mRNA may result in cognitive impairment, especially attention deficit in patients with schizophrenia, possibly through triggering a cascade of inflammatory responses in frontal cortex.

Some limitation in this study should be mentioned. First, we evaluated the *NLRP3* mRNA in peripheral level, not in brain. It is uncertain whether the peripheral level of *NLRP3* mRNA is correlated with the level in the central nervous system. Second, the lack of a significant association of rs10754558 polymorphism and schizophrenia may be caused by the modest sample size, whereas the power reached statistical significance. Accordingly, our findings should be considered only preliminary and require further investigations for validation in independent samples. Third, this study employed samples from Eastern China and may not be representative of the Han Chinese population in general, nor other closely related populations in the area. Finally, we have analyzed only one functional SNP in *NLRP3* to evaluate the genetic relationship with schizophrenia. This means further analyses need to be carried out with more variants in the *NLRP3* for validation of our results.

In conclusion, we performed a comprehensive analysis to detect the association of *NLRP3* with schizophrenia in Han Chinese. Although our findings did not support *NLRP3* confer susceptibility to schizophrenia, *NLRP3* may be a risk factor for cognitive impairment, especially attention deficit in this disorder. Further investigations are required for validation of our results in larger and independent sample across various ethnicities.

## Data Availability

The original contributions presented in the study are publicly available. This data can be found here: https://datadryad.org/stash/share/vfrPQUqJNc28WuM76t41oWjYIBJY403RcXSMTr33pjM
