# Supplementary material for: NLRP3 Influences Cognitive Function in Schizophrenia in Han Chinese
Source: Front Genet. 2021 Dec 10;12:781625. doi: 10.3389/fgene.2021.781625 (PMC8702823; doi:10.3389/fgene.2021.781625)
Supplement: Supplementary file 1 [file DataSheet1.doc]

**Supplementary Table S1. Demographics of schizophrenia and control groups for expression analyses**

|  | Cases | Controls | *P* |
| --- | --- | --- | --- |
| Number of subjects (n) | 53 | 56 |  |
| Age (years), mean (SD) | 30.06 (5.66) | 29.13 (5.86) | 0.25 |
| Gender, male n (%) | 28 (54.9) | 33 (56.9) | 0.40 |
| Smoking status, n (%) | 14 (26.4) | 8 (14.3) | 0.15 |
| Duration of illness (month) ^a^, mean (SD) | 8.67 (3.22) | NA |  |

Note: ^a^ Duration of illness prior to admission

**Supplementary Figure S1. Differential expression of *NLRP3* mRNA in brain between patients with schizophrenia and healthy controls.** Each bar represents the average level of *NLRP3* expression. Error bars represent the standard deviation of the mean value. Data was extracted from the SZDB database (<http://www.szdb.org/>).

**
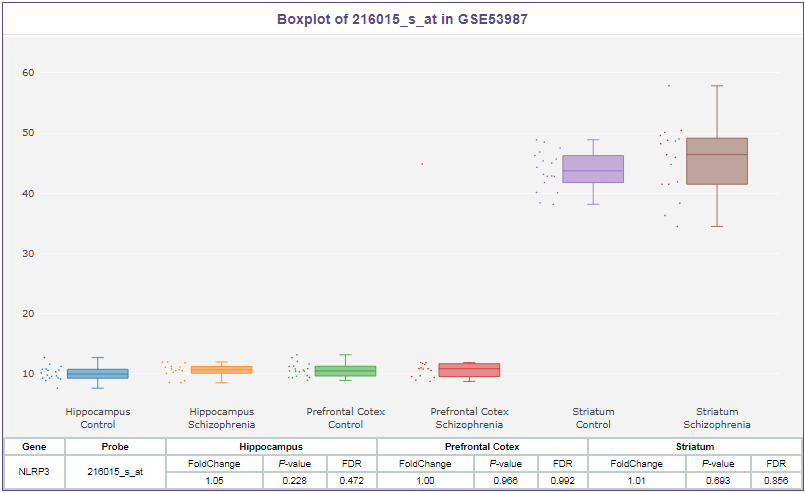
**

**Supplementary Figure S2. Association of rs10754558 with schizophrenia in PGC database**
